# Theoretical and Experimental Investigations into a Crawling Robot Propelled by Piezoelectric Material

**DOI:** 10.3390/mi12121577

**Published:** 2021-12-18

**Authors:** Xiangli Zeng, Yue Wu, Shangyan Han, Yanbo Liu, Haohua Xiu, Fengjun Tian, Luquan Ren

**Affiliations:** 1 School of Mechanical and Aerospace Engineering, Jilin University, Changchun 130025, China; xlzeng17@mails.jlu.edu.cn (X.Z.); wuyue@jlu.edu.cn (Y.W.); hansy20@mails.jlu.edu.cn (S.H.); liuyb19@mails.jlu.edu.cn (Y.L.); tianfj@jlu.edu.cn (F.T.); 2 Key Laboratory of Bionic Engineering (Ministry of Education, China), Jilin University, Changchun 130025, China; lqren@jlu.edu.cn

**Keywords:** piezoelectric actuator, flexible robot, millimetre-sized robot, functional material

## Abstract

Conventional motors with complicated electromagnetic structures are difficult to miniaturise for millimetre- and centimetre-sized robots. Instead, small-scale robots are actuated using a variety of functional materials. We proposed a novel robot propelled by a piezoelectric ceramic in this work. The robot advances due to the asymmetric friction created by the spikes on the surface. The structural modelling was completed, static and dynamic models were established to predict the moving characteristics, the prototype was built using three dimensional (3D) printing technology, and the models were evaluated via experiments. Compared with conventional inchworm-type robots, the proposed robot is superior in simple structure because the clamping components are replaced by spikes with asymmetric friction. Compared with SMA (shape memory alloy) actuating inchworm-type robots, it has a faster velocity with higher resolution. Meanwhile, the components are printed through an additive manufacturing process that is convenient and avoids assembly errors. This design could make contributions to many areas, such as pipe inspection, earthquake rescue, and medicine delivery.

## 1. Introduction

Robots inspired by inchworms mimic the motion principle of a natural inchworm. To finish the distance accumulation, inchworm-type robots move step by step. This kind of stepping motion makes up the application of the piezoelectric actuator of which the deformation is too small to drive things. In a review from Jianping Li et al. [1], the principles of piezoelectric actuators mimicking inchworms can be divided into three kinds of mechanisms: the “walker” [2], the “pusher” [3], and the hybrid “walker–pusher” [4,5]. However, almost all inchworm type piezoelectric actuators require three piezoelectric units [6,7,8,9]: two for clamping and one for driving, which makes their structure and controls complicated. Tian X et al. presented an actuator that achieves clamping and driving by different resonance models of the structure [10]. Nevertheless, it demands three piezoelectric chips, too.

In practice, a number of robots based on inchworms were proposed besides those employing piezoelectric actuators. Pneumatic actuators, EPA (electroactive polymer), liquid elastomer, and SMA (shape memory alloy) are among the functional materials applied for driving. Ahmad Rafsanjani et al. designed a pneumatic snake-shaped robot using origami technology to achieve asymmetric friction [11]. E.F. Markus Henke et al. demonstrated a robot could transform the electromechanical oscillations generated by the EPA in the plane into crawling motion [12]. Chengjun Wang et al. used resistance wires to heat a liquid elastomer to mimic inchworms with three segments [13]. Huai Ti Lin et al. proposed a soft robot GoQBot powered by SMA [14]. Takuya Umedachi et al. presented a robot moving through asymmetric friction which was formed by two types of material [15]. Robots driven by functional materials have a much simpler structure due to their asymmetric friction. Nevertheless, the common issue is slow movement speed.

Rather than the absolute clamping controlling of inchworm-type piezoelectric actuators, the robots mentioned took advantage of the deviation between asymmetric frictions. It is known that friction changes when the shape, material, and pressure vary. In nature, Galium aparine, gecko feet, eupatoria and lutetiana, snake skin, and flatfish skin are all proven to have anisotropic friction [16]. Paul Day et al. offered more proof that the spikes could lead to asymmetric friction [17]. Therefore, we used the same method to propel the designed robot. Even though it could not be controlled precisely without complete clamping units, the robot has a much simpler structure, more available controlling signal, and rapid movement. 

In this research, the modelling and theoretical analysis were completed. In the process of modelling, COMSOL software was applied to determine the proper resonance frequency for the piezoelectric actuator. Then, mathematical calculations were performed for the motion prediction of the robot. Following that, a set of experiments were conducted to explore the influence of the structure parameters. In the end, the theoretical and experimental results were compared to verify the model of the robot. Table 1 indicates that, compared with previous inchworm-type piezoelectric actuators, the proposed robot has a simpler structure and the stimulation frequency is more easily acquirable. Moreover, the moving speed exceeds that of robots powered by other functional materials. 

## 2. Modelling and Theoretical Analysis

### 2.1. Modelling

**Figure 1:**
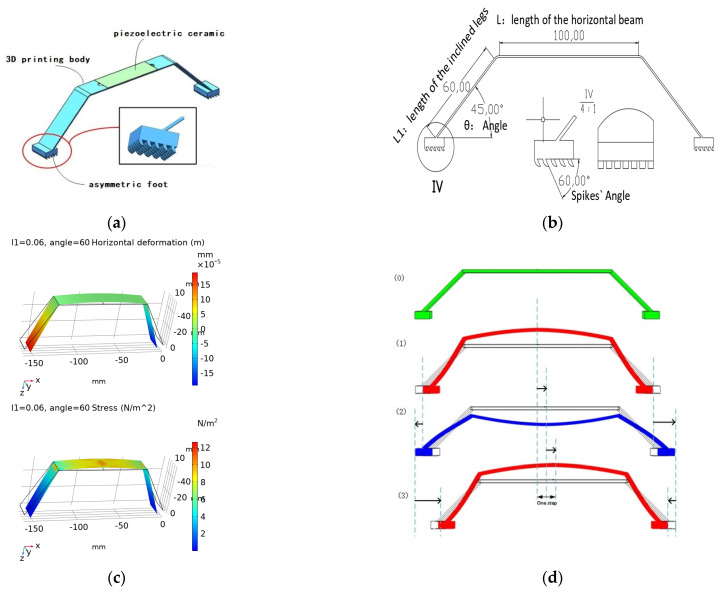
(**a**) The structure of the robot, (**b**) parameters of the microstructures (spikes), (**c**) first-order resonant model, (**d**) locomotion process.

This flexible robot comprises a piezoelectric ceramic (Table 2 shows parameters), a 3D-printed body, and asymmetric feet (feet with asymmetric friction) (Figure 1a). The asymmetric feet contact the working plane with microstructures that produce asymmetric friction. Under voltage stimulation, the piezoelectric ceramic bend, and the bending deformation powers the robot. The electric energy flows from piezoelectric ceramic to the printed body and finally forms the moving ability due to the asymmetric friction.

Due to low cost and convenient fabrication, the body part and spikes on the feet were fabricated using three dimensional (3D) printing (Material: 8200 resin; Poisson’s ratio 0.4–0.44; density 1.12~1.18 g/cm^3^; Young‘s modulus 2692–2775 MPa, WeNext Technology Co., Ltd. Shengzhen, China). The piezoelectric ceramic was glued to the horizontal beam. Here, the microstructures (spikes on the feet) are essential for asymmetric friction and motion. The spikes (shown in Figure 1b) are excised from a cube (2 mm × 2 mm × 2 mm). The spike angle (60° in the figure) can be changed for varied asymmetric friction. As shown in Figure 1b, more friction appears when the spike part moves to the right and less friction appears when it moves to the left. 

In COMSOL Multiphysics^©^ 5.6 (COMSOL Inc., Stockholm, Sweden), we can obtain the proper resonance frequency by model analysis. Figure 1c shows the first-order resonance states in which the colour indicates deformation or stress. There is one pair of positions that has the most significant horizontal deformation. Therefore, the first-order resonance model was chosen as the working status. The specific parameters are stated in Table 3. Figure 1d explains the moving process. In Figure 1d, status 0 means the original shape, status 1 and 3 indicate the conditions when the voltage reaches the peak, and status 2 means the valley. When the voltage moves from the peak to the valley (status 1 to 2), the centre of the 3D-printed body would move downward, which could propel the feet to depart. The right foot would move forward with lower friction, but the left one would move backwards with higher friction. Synthetically, the resultant friction is supposed to propel the robot moves forward. However, from status 2 to 3, when the voltage is from the valley to the peak, the feet approach each other. Similarly, the right foot moves backwards with higher friction, but the left one moves forwards with smaller friction. The resultant friction keeps propelling the robot. In the end, the robot crawls forward, step by step. 

### 2.2. Theoretical Analysis

To detect the movement pattern and predict the characteristics of the robot, static and kinetic models were established and analysed for a piezoelectric ceramic whose size is l×b×h, when it is stimulated by the voltage of $U=U_{0}sin(\omega t$*)*. The infinitesimal piezoelectric ceramics could thus be analysed. Here, the second-order piezoelectric equation was applied in that the voltage (z-direction) is vertical to the deformation (x-direction) [18].

(1) $$\frac{\partial \sigma_{x}\left(x,t\right)}{\partial x}-\rho \frac{\partial^{2}\sigma_{x}\left(x,t\right)}{\partial t^{2}}=0$$

(2) $$\{\begin{array}{c} \sigma_{x}=c_{11}^{E}S_{1}-e_{31}E_{3}\\ D_{3}=e_{31}S_{1}+\varepsilon_{33}^{S}E_{3} \end{array}$$

where

σ is the stress in PZT, 

ρ is the density of PZT,

$c_{11}^{E}$ is short circuit elastic stiffness coefficient, 

$S_{1}$ is the strain in PZT,

$e_{31}$ is piezoelectric stress constant, 

$E_{3}$ is electric field intensity, 

$D_{3}$ is the electric displacement, and

$\varepsilon_{33}^{S}$ is the clamping voltage constant. 

The equivalent shear force *Q* transmitted by the piezoelectric wafer to the matrix through the adhesive layer and its position *x* is expressed as follows:

(3) $$\{\begin{array}{l} Q=\frac{G_{j}\Lambda b}{\gamma^{2}h_{j}}\left[sec\left(\frac{\gamma l}{2}\right)-1\right]sin\left(\omega t\right)\\ l_{0}=\pm \frac{l\left[sin\left(\frac{\gamma l}{2}\right)-\gamma cos\left(\frac{\gamma l}{2}\right)\right]}{2\gamma \left[1-cos\left(\frac{\gamma l}{2}\right)\right]} \end{array}$$

$$\Lambda =d_{31}\frac{U_{0}}{h}\;\gamma =\sqrt{\frac{\rho hh_{j}\omega^{2}+G_{j}}{c_{11}^{E}hh_{j}}}$$

where

$G_{j}$ is the shear modulus of the adhesive layer,

$h_{j}$ is the thickness of the adhesive layer, and

$d_{31}$ is the piezoelectric constant.

According to the calculated shear force and its position, the driving force received by the 3D-printed body can be equivalent to a pair of bending moments. The horizontal displacement at the end of the robot can be calculated.

(4) $$x=\int_{0}^{l_{0}}\left(\frac{\delta^{2}}{2}\right)dx+\int_{l_{0}}^{\frac{L}{2}}\left(\frac{\delta^{2}}{2}\right)dx+\int_{\frac{L}{2}}^{\frac{LR}{2}}\left(sin\theta *\delta -\frac{cos\theta }{2}*\delta^{2}\right)dx$$

(5) $$\delta \left(x\right)=\{\begin{array}{c} \frac{1}{EI}\left(\frac{Mx^{3}}{3LR}+C\right)0\leq x<l_{0}\\ \frac{1}{EI}\left(\frac{Mx^{3}}{3LR}-\frac{M}{2}{\left(x-l_{0}\right)}^{2}+C\;\right)l_{0}\leq x<\frac{LR}{2} \end{array}$$

$$h_{j}\;LR=L+2L_{1}\cos\theta$$

where

E is Young’s modulus of the 3D-printed body,

I is the moment of inertia of the 3D-printed body,

L is the length of the horizontal beam,

$L_{1}$ is the length of the inclined legs, and

θ is the angle of the inclined legs.

**Figure 2:**
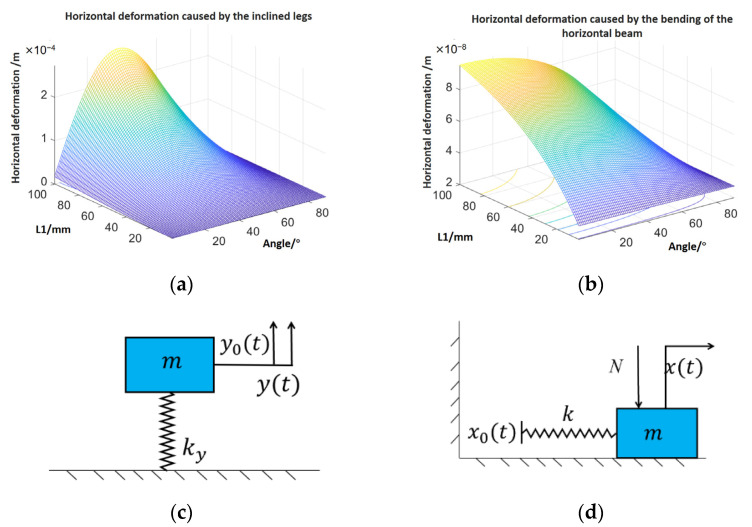
(**a**) The horizontal deformation caused by the inclined legs, (**b**) the horizontal deformation caused by the bending of the horizontal beam, (**c**) the vertical dynamic model, (**d**) the horizontal dynamic model.

The horizontal deformation consists of two parts: one is the bending of the horizontal beam, the other occurs because of the inclined legs. In Figure 2a,b, it is obvious that the deformation caused by the legs is much larger than the bending. The results of Equation (1) are employed as the horizontal input ($x_{0}\left(t\right)$) in the dynamic model in Figure 2d.

The dynamic model is shown in Figure 2. The vertical input $y_{0}\left(t\right)=psin\left(\omega t\right)$ is the deformation of the piezoelectric ceramic, and the kinetic equation is as follows.

(6) $$-k_{y}\left(y\left(t\right)+y_{0}\left(t\right)\right)=m\ddot{y\left(t\right)}$$

where $k_{y}$ means the stiffness in the vertical dimension and y(t)=qsin(ωt) is a particular solution to the differential equation. In this situation, the output could be solved as

(7) $$y\left(t\right)=-\frac{k_{y}psin\left(\omega t\right)}{k_{y}+m\omega^{2}}$$

Similarly, the horizontal deformation could be derived as follows.

(8) $$k\left[x_{0}\left(t\right)-x\left(t\right)\right]-f=m\ddot{x\left(t\right)}$$

$$f=\{\begin{array}{c} \mu_{1}N\dot{x\left(t\right)}>0\\ \mu_{2}N\dot{x\left(t\right)}<0 \end{array}k=\frac{Ebh}{L+2L_{1}cos^{2}\theta +2hsin^{2}\theta }$$

Once we let $x_{0}\left(t\right)=A_{2}\sin\left(\omega t\right),\;f/k=\mu_{1}k_{y}y\left(t\right)/k=A_{1}\sin\left(\omega t\right),\;\mathrm{and}\;f/k=\mu_{2}k_{y}y\left(t\right)/k=A_{1}^{\prime }\sin\left(\omega t\right)$, then the deformation equation of the crawling robot can be deduced.

$$x\left(t\right)=A\sin\left(\omega t\right)=\frac{k_{1}\left(A_{2}-A_{1}\right)}{k_{1}-\omega^{2}m}\sin\left(\omega t\right)$$

$$x\left(t\right)=A^{\prime }\sin\left(\omega t\right)=\frac{k_{1}\left(A_{2}-A_{1}^{\prime }\right)}{k_{1}-\omega^{2}m}\sin\left(\omega t\right)$$

(9) $$Step=A-A^{\prime }\;$$

where

$\mu_{1}$ is the friction coefficient when the robot moves forwards and

$\mu_{2}$ is the friction coefficient when the robot moves backwards.

**Figure 3:**
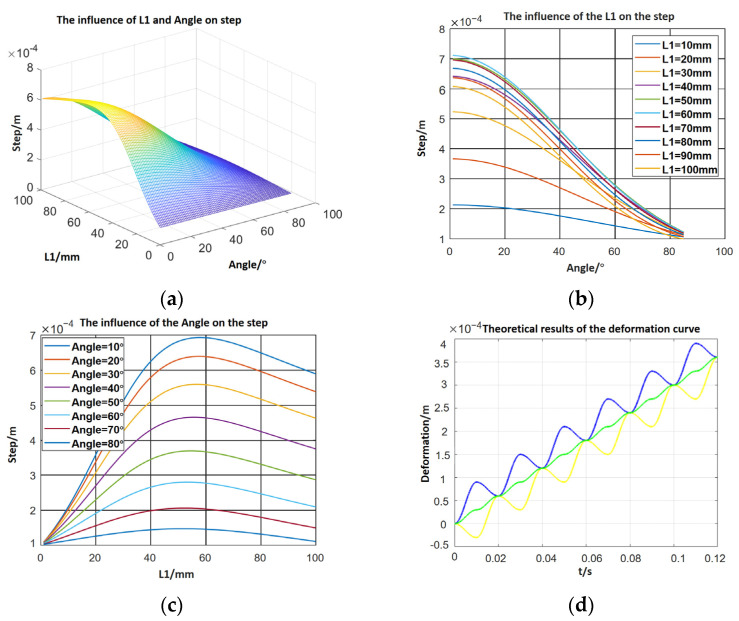
(**a**) The relationship between step, leg length and angle; (**b**) the relationship between step and leg length; (**c**) the relationship between step and angle; (**d**) the relationship between deformation and time.

Hence, the influence of the length and the inclined leg angle can be displayed, as shown in Figure 3. In Figure 3a–c, the length and angle of the inclined legs influence the step directly. In specific, the step decreases when the angle increases and the step reaches the maximum when the length equals 60 mm. Meanwhile, the deformation curves of the left and right foot and the mass centre could be obtained.

(10) $$x_{r}\left(t\right)=\{\begin{array}{ll} Asin\left(\omega t-\frac{\pi }{2}\right)+A & ,0\leq t<\frac{T}{2}\\ A^{\prime }sin\left(\omega t-\frac{\pi }{2}\right)-A^{\prime }+2A & ,\frac{T}{2}\leq t<T \end{array}$$

(11) $$x_{l}\left(t\right)=\{\begin{array}{ll} A^{\prime }sin\left(\omega t+\frac{\pi }{2}\right)-A^{\prime } & ,\;0\leq t<\frac{T}{2}\\ Asin\left(\omega t+\frac{\pi }{2}\right)+A-2A^{\prime }\; & ,\;\frac{T}{2}\leq t<T \end{array}$$

(12) $$x\left(t\right)=\frac{x_{r}\left(t\right)+x_{l}\left(t\right)}{2}$$

In Figure 3d, the blue line means the deformation of the right foot, the yellow means the left foot, and the green one means the centre of the mass. It is obvious that the deformation formulas of two ends are a curve that overlaps a sinusoidal and a linear function. 

## 3. Experimental Analysis and Discussion

### 3.1. Experimental Analysis

#### 3.1.1. The Influence of the Microstructure on Feet

As the spike angle produces asymmetric friction, it is necessary to figure out the friction coefficient for different angles and directions. Spikes with different angles were printed to test the influence of the microstructure on the foot. 

**Figure 4:**
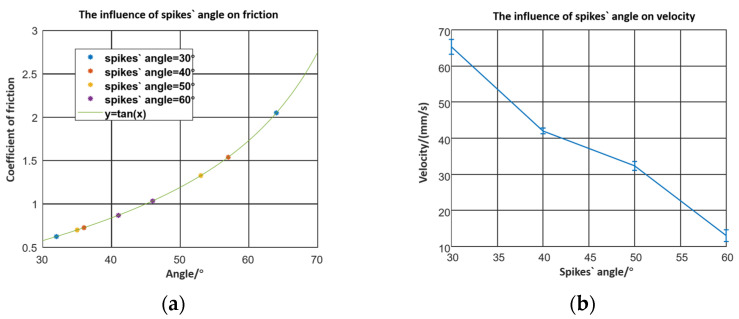
(**a**) The friction factors of different inclined angles; (**b**) the influence of the inclined angle between microstructures and the horizontal direction.

In Figure 4a, the friction factor could be calculated by the friction angle. In the testing method, the robot is put on the working plane and the plane slopes gradually. The friction angle is the working plane’s angle at which the crawling robot begins to slide to the right. Experimentally, the friction factors for different spike angles were measured when the robot was put forward and reversed. When the spikes with angles of 30°, 40°, 50°, and 60° were placed forward, the friction coefficients between the flexible robot and the working plane were 0.6249, 0.7265, 0.7002, and 0.8693, respectively. Meanwhile, the friction coefficients were 2.05, 1.54, 1.327, and 1.036 respectively when the robots reversed. The deviations between the two directions were 1.4251, 0.8135, 0.6268, and 0.1667. The larger the deviation, the faster the robot moved. As shown in Figure 4a, the friction coefficient of the backwards direction increases sharply, but in the forward direction, it decreases smoothly when the spike angle becomes smaller.

Figure 4b shows that the robot moves slowly and when the spike angle increases. This behaviour is explained by Equation (9). The difference values of the backwards and forwards friction coefficients determine the velocity of the robot. 

#### 3.1.2. The Influence of the Structural Parameters

**Figure 5:**
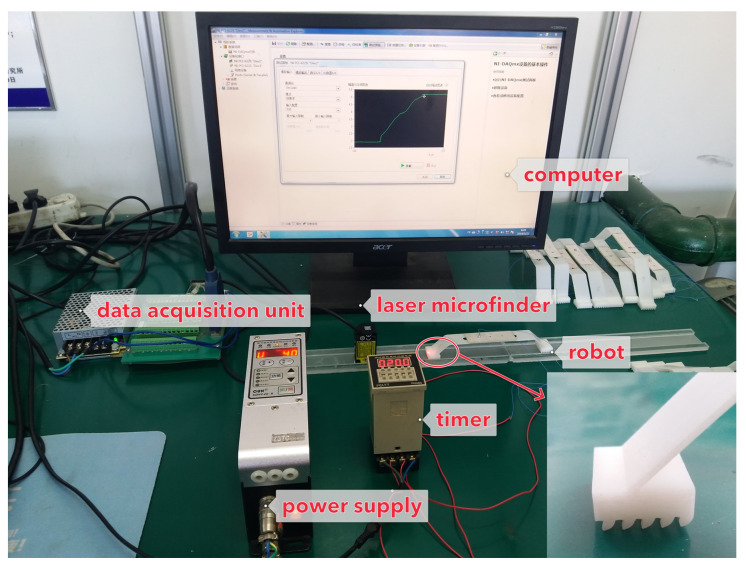
The experimental instruments.

The structural parameters of the inclined leg’s length and angle have a significant influence on the motion characteristics. Therefore, sixteen groups of robots with varying lengths and angles were fabricated by 3D printing. The length values were 40 mm, 50 mm, 60 mm, and 70 mm, respectively, and the angles were 30°, 45°, 60°, and 75°, respectively. In Figure 5, the power is output by a signal generator (RIGOL-DG4062) with an amplifier. The deformation of the foot was measured by a laser rangefinder. When the crawling robot is stimulated by electric power, the robot would move forward and the laser microfinder would capture the deformation data. 

The piezoelectric actuators always work on resonance, and the resonance frequency would alter once the structure parameters changed. Therefore, when exploring the influence of structural parameters, it is necessary to power the robot at different frequencies. 

**Figure 6:**
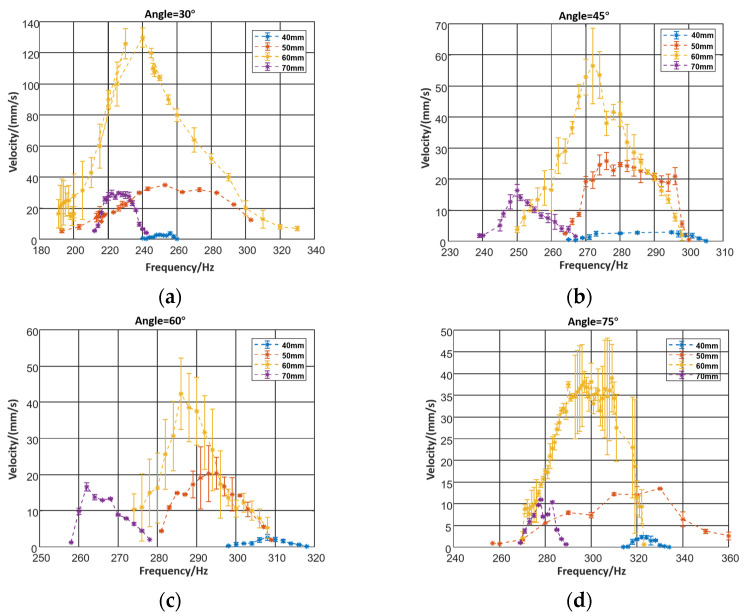
(**a**) Relationship between velocity and leg length when leg angle = 30°, (**b**) relationship between velocity and leg length when leg angle = 45°, (**c**) relationship between velocity and leg length when leg angle = 60°, (**d**) relationship between velocity and leg length when leg angle = 75°.

Figure 6 shows how the effect of the leg length and angle was determined. The peak of the frequency–velocity curve indicates that the system is at resonance. The velocity increases at the beginning and decreases later when the length of the leg becomes longer. Due to the flexible deformation of the robot’s legs, the velocity of the robot increases when the length elongates. However, the legs‘ elastic deformation absorbs the deformation which might diminish the moving distance of each foot, and weaken the robot’s motion ability, making prediction more difficult.

**Figure 7:**
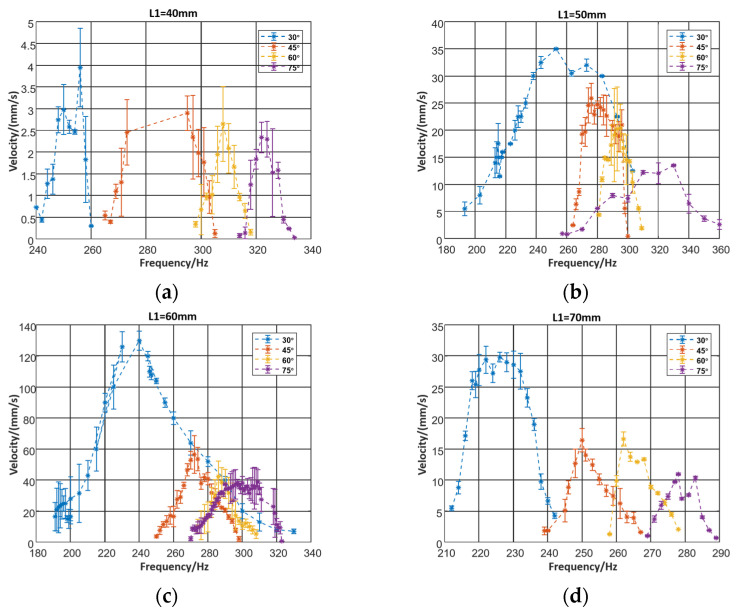
(**a**) Relationship between velocity and leg angle when leg length = 40 mm (**b**) relationship between velocity and leg angle when leg length = 50 mm, (**c**) relationship between velocity and leg angle when leg length = 60 mm, (**d**) relationship between velocity and leg angle when leg length = 70 mm.

In the same manner, as we can see in Figure 7, the speed increases when the angle decreases. Mainly, the velocity of the robot gets slower if the angle becomes bigger. In Equation (4), we found that the input of the horizontal dynamic model $x_{0}\left(t\right)$ is partly caused by the inclined legs. 

The robot with the leg length of 60 mm and an angle of 30° moved fastest. This could be explained by the equivalent stiffness in the horizontal plane. The increase of the length and the decrease of the angle would cause a reduction of the stiffness. Meanwhile, the stiffness would positively impact the resonance frequency, which diminishes gradually when the peak of the velocity–frequency curve appears.

#### 3.1.3. The Influence of the Powering

**Figure 8:**
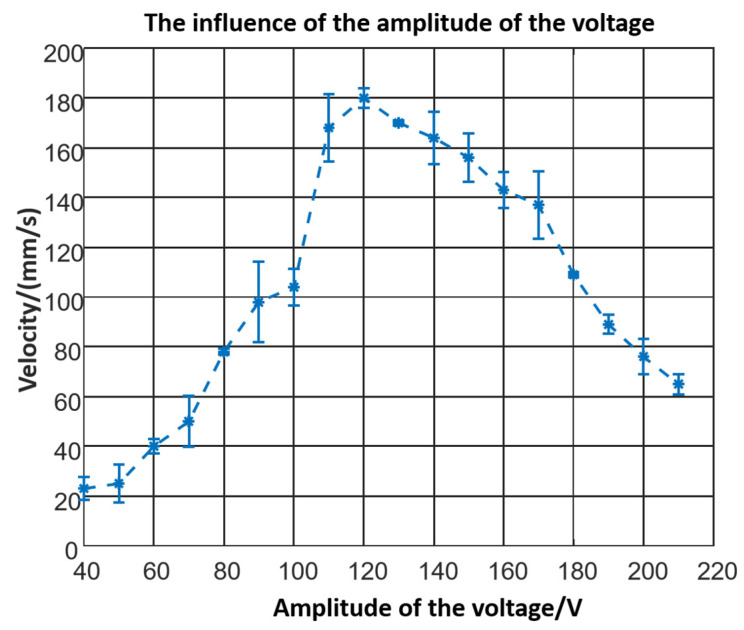
The influence of the voltage.

The signal generator could adjust the amplitude of the voltage from 0 to 400 V. Using the 60 mm prototype with a 30° leg, the influence of the actuating voltage could be tested. Voltage smaller than 40 V could not actuate the robot due to the threshold. The robot moves faster as the voltage increases from 40 to 120 V. However, the velocity decreases while the voltage increases sequentially, as shown in Figure 8. In the experiment, the robot began to jump, and the collision consumed energy. 

**Figure 9:**
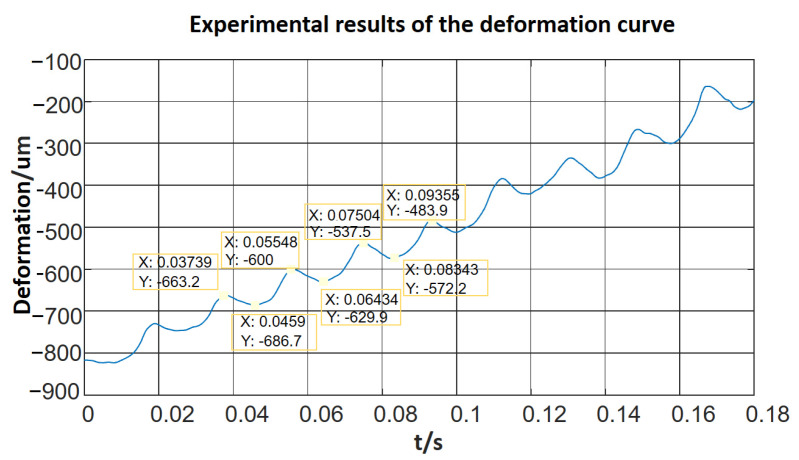
Deformation curve of one end of the robot.

Finally, the laser rangefinder was used to test foot deformation, and the results are shown in Figure 9. Each foot deformed step-by-step. According to the experimental data, the positive step equalled 89.13 mm/s and the negative step equalled 29.4 mm/s. In the end, the mean step was 59.73 mm/s. The step(resolution) was defined as the deformation under one cycle sinusoidal period of stimulation and the velocity was defined as all steps in one second.

### 3.2. Discussion

**Figure 10:**
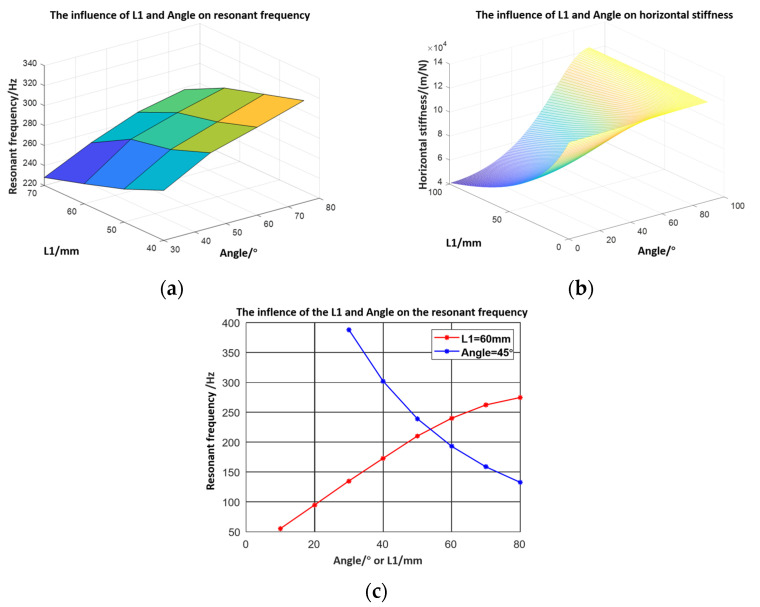
(**a**) Experimental resonance frequency data of robots with varied structure parameters; (**b**) stiffness of robots with varied structure parameters; (**c**) influence of L1 and angle on the resonance frequency in simulation.

The experimental resonance frequency data of robots with varied structure parameters is shown in Figure 10a. The resonance frequency changes as the stiffness changes. Compared with the stiffness tendency shown in Figure 10b, it could be concluded that the experimental results meet the theoretical results. In Figure 10c, the resonance frequency with varied inclined leg‘s length and angle could be calculated by COMSOL simulation (the values are all amplified because the piezoelectric ceramic and glue are ignored in the simulation). However, it is obvious that the trend meets the experimental results.

Moreover, according to the frequency–velocity relationship of the piezoelectric actuator, the velocity increases dramatically when the system is resonant. The theoretical effect was matched by the experimental results. At the same time, the deformation curve described by the theory in Figure 3b was consistent with the experimental results shown in Figure 9. The experiments validate the theoretical model.

## 4. Conclusions

Based on piezoelectric beam theory and dynamic theory, a crawling robot propelled by a piezoelectric ceramic was proposed, and static and kinetic analyses were completed. Key structure parameters were researched, and the velocity characteristics were tested. The robot would move slowly when the angle increases and run fastest when the length of the leg reaches 60 mm. The robot should work in resonant. However, the velocity would decrease once the amplitude of the voltage overcomes a threshold. The robot could crawl at a speed of 178.68 mm/s when it was powered by a voltage of 120 V. The resolution of the robot is 60 μm. Meanwhile, compared with the conventional inchworm-type piezoelectric actuator, the robot has a simple structure and a fetchable power signal. Compared with inchworm-inspired robots actuated by other functional materials like SMA, the proposed robot could move much faster due to the quick response and high-frequency stimulation of the piezoelectric chip. 

## Figures and Tables

**Table 1:** Characteristics of crawling robots.

Powering Material	Size (mm × mm × mm)	Velocity (mm/s)	Stimulation
Piezoelectric [5]	100.6 × 74.6 × 7	1641	100 V 41.35 kHz
Pneumatic [6]	164 × 25 × 25	6	30 kPa
EPA [7]	250 × 8 × -	1	4 kV
Laser [8]	30 × 20 × 10	0.032	60 °C
SMA [9]	10 × - × -	0.2	15 W
Piezoelectric (in this article)	202 × 20 × 30	178.68	120 V 240 Hz

**Table 2:** Parameters of piezoelectric ceramic.

Parameters	Company	Density	Short Circuit Elastic Stiffness Coefficient	Piezoelectric Stress Constant	Clamping voltage Constant
Value	Harbin Rongzhi Naxin Technology Co., Ltd.	7800 kg/m^3^	131.1 Gpa	−5.2 C/m	5.63 × 10^−9^ F/m

**Table 3:** Structure parameters of the robot.

Parameters	Value
Piezoelectric Ceramic(length × width × height)	60 × 20 × 0.2 (mm × mm × mm)
3D-printed body(width × height)	20 × 0.2 (mm × mm)
L: Length of the horizontal beam	100 (mm)
L1: Length of the inclined legs	40/50/60/70 (mm)
θ: Angle of the inclined legs	30/45/60/75 (°)
Spike angle	30/40/50/60 (°)
